# Assessing the Robustness of Mediation Analysis Results Using Multiverse Analysis

**DOI:** 10.1007/s11121-021-01280-1

**Published:** 2021-07-16

**Authors:** Judith J. M. Rijnhart, Jos W. R. Twisk, Dorly J. H. Deeg, Martijn W. Heymans

**Affiliations:** Department of Epidemiology and Data Science, Amsterdam UMC, Location VU University Medical Center, Amsterdam Public Health Research Institute, Amsterdam, The Netherlands

**Keywords:** Multiverse analysis, Reproducibility, Robustness, Specification curve, Selective reporting, Transparency, Mediation analysis, Indirect effect

## Abstract

There is an increasing awareness that replication should become common practice in empirical studies. However, study results might fail to replicate for various reasons. The robustness of published study results can be assessed using the relatively new multiverse-analysis methodology, in which the robustness of the effect estimates against data analytical decisions is assessed. However, the uptake of multiverse analysis in empirical studies remains low, which might be due to the scarcity of guidance available on performing multiverse analysis. Researchers might experience difficulties in identifying data analytical decisions and in summarizing the large number of effect estimates yielded by a multiverse analysis. These difficulties are amplified when applying multiverse analysis to assess the robustness of the effect estimates from a mediation analysis, as a mediation analysis involves more data analytical decisions than a bivariate analysis. The aim of this paper is to provide an overview and worked example of the use of multiverse analysis to assess the robustness of the effect estimates from a mediation analysis. We showed that the number of data analytical decisions in a mediation analysis is larger than in a bivariate analysis. By using a real-life data example from the Longitudinal Aging Study Amsterdam, we demonstrated the application of multiverse analysis to a mediation analysis. This included the use of specification curves to determine the impact of data analytical decisions on the magnitude and statistical significance of the direct, indirect, and total effect estimates. Although the multiverse analysis methodology is still relatively new and future research is needed to further advance this methodology, this paper shows that multiverse analysis is a useful method for the assessment of the robustness of the direct, indirect, and total effect estimates in a mediation analysis and thereby to inform replication studies.

**Supplementary Information:**

The online version contains supplementary material available at 10.1007/s11121-021-01280-1.

## Introduction

In the last two decades, various reports have been published that stated that a substantial number of published study results cannot be replicated (Ioannidis, 2005; Open Science Collaboration, 2015). These reports caused an increased awareness of the importance of replication studies among researchers from various research fields, including psychology and epidemiology (Anderson & Maxwell, 2016; Lash et al., 2018; Valentine et al., 2011). Replication studies aim to replicate the original study results in a new sample using the same research methodology as in the original study (Goodman et al., 2016). However, published study results might fail to replicate for various reasons, including questionable research practices (QRPs) and researcher degrees of freedom (RDFs) (Anderson & Maxwell, 2016; Wicherts et al., 2016). QRPs are practices that increase the chances of finding results that are in line with the research hypotheses, such as selective reporting, and RDFs are the arbitrary choices that researchers make when analyzing the data (Fiedler & Schwarz, 2016; Wicherts et al., 2016). When published study results are robust against these RDFs, then replication studies are more likely to reproduce the published study results (Nuijten et al., 2018).

To avoid wasting resources, Nuijten et al. (2018) suggested to first reproduce the published results using the original data and then verify the robustness of published results against data analytical decisions before conducting a replication study. When preparing and analyzing data, researchers are faced with various data analytical decisions. These decisions may be study-centric (e.g., exclusion criteria and missing data handling), variable-centric (e.g., variable transformations), or model-centric (e.g., the inclusion of interactions, random effects, and covariates in the statistical model). Because these decisions are often arbitrary and multiple reasonable decisions can be made, the decisions are referred to as the “garden of forking paths” (Gelman & Loken, 2013). The reported model is only one of the many reasonable models that could have been estimated based on the raw data.

Data analytical decisions are made based on various reasons, such as the theoretical and statistical validity of the model, methodology used in prior studies, data constraints, limited statistical expertise, ease of communicating the effect estimates, and the belief that alternative analyses would have little impact on the results (Kale et al., 2019; Liu et al., 2020). The subjectivity in data analytical decisions was demonstrated by Silberzahn et al. (2018), who asked 29 research teams to answer the same research question using the same dataset. This resulted in 29 different statistical analyses, with variations in the set of covariates and in the statistical modeling approach, which ranged from simple linear regression to Bayesian analyses. Therefore, the analyses resulted in 29 different effect estimates. The variation in the effect estimates could not be explained by differences in statistical expertise or by peer-ratings of the quality of the analyses (Silberzahn et al., 2018).

In some situations, researchers acknowledge the subjectivity in the data analytical decisions by performing multiple analyses, but opt to report only one of the acquired results (Kale et al., 2019; Liu et al., 2020). Some researchers deem the reporting of only one result sufficient when all research results point in the same direction (Liu et al., 2020). When the research results point in various directions, researchers sometimes choose to only report statistically significant results that are in line with their hypotheses, which is also known as *p*-hacking (Gelman & Loken, 2013). Other reasons for selective reporting are feeling the need to tell a clear story, the anticipation that reviewers or colleagues in the field will disapprove of certain data analytical decisions, and journal constraints on the length of a paper (Kale et al., 2019; Liu et al., 2020).

To increase the transparency in the impact of data analytical decisions on the effect estimates and to avoid selective reporting, it has been suggested to report effect estimates based on all reasonable data analytical decisions (Nuijten et al., 2018; Silberzahn et al., 2018; Steegen et al., 2016). This has been referred to as a multiverse analysis (Steegen et al., 2016), specification curve analysis (Simonsohn et al., 2020), vibration of effects (Patel et al., 2015), or multi-model analysis (Young & Holsteen, 2017). In contrast with conventional sensitivity analyses, which often include a limited set of alternative data analytical decisions selected by the researcher, a multiverse analysis aims to identify all decision points and perform the analyses across *all* reasonable alternative decisions (Simonsohn et al., 2020; Steegen et al., 2016). Therefore, a multiverse analysis provides insight into all combinations of data analytical decisions leading to effect estimates that either support or contradict the research hypothesis. For example, the multiverse analysis performed by McBee et al. (2019) showed that the statistical significance of an earlier reported association between TV watching in early childhood and attention problems in later childhood was highly dependent on the cut-off point chosen for the binary attention problems variable.

Although multiverse analysis has the potential to contribute to the acquisition of reliable knowledge, the little available guidance might prevent researchers from applying multiverse analysis (Dragicevic et al., 2019). Researchers may experience difficulties in defining the multiverse (Liu et al., 2020), and in summarizing and interpreting the large number of effect estimates yielded by the multiverse analysis (Dragicevic et al., 2019). These difficulties are amplified when performing a multiverse analysis of more complex models, such as mediation models. Mediation analysis is often applied in prevention research to decompose the total determinant-outcome effect estimate into an indirect effect estimate through a mediator variable, and a direct effect estimate (Judd & Kenny, 1981; MacKinnon, 2008). For example, Jackson et al. (2016) used mediation analysis to assess the intermediate effects of an intervention consisting of a parenting program on the susceptibility of schoolchildren to alcohol use, and Kwok and Gu (2019) used mediation analysis to assess whether adolescents’ depressive symptoms mediated the relation between childhood neglect and adolescent suicidal ideation.

Due to the addition of a mediator variable and the estimation of multiple effects, researchers face more variable-centric and model-centric data analytical decisions that could impact the study results than in a bivariate analysis, i.e., a simple determinant-outcome analysis. As a result, the potential multiverse of a mediation analysis is larger than the potential multiverse of a bivariate analysis. For example, there might be reasonable alternative operationalizations of the mediator variable, and confounders and moderators need to be considered for each of the effect estimates in the mediation model (MacKinnon, 2008). These data analytical decisions may not only impact the magnitude and statistical significance of the total determinant-outcome effect estimate, but also the magnitude and statistical significance of the direct and indirect effect estimates.

The aim of this paper is to provide an overview and worked example of the use of multiverse analysis to assess the robustness of the effect estimates from a mediation analysis. We first provide a brief introduction into mediation analysis. We then summarize the multiverse analysis literature and describe how multiverse analysis can be used to assess the robustness of the effect estimates yielded by mediation analysis. Subsequently, we demonstrate the multiverse analysis of a published mediation analysis using a real-life data example from the Longitudinal Aging Study Amsterdam. Finally, we discuss the strengths and limitations of multiverse analysis and provide recommendations for future methodological research on multiverse analysis.

## Mediation Analysis

**Fig. 1:**
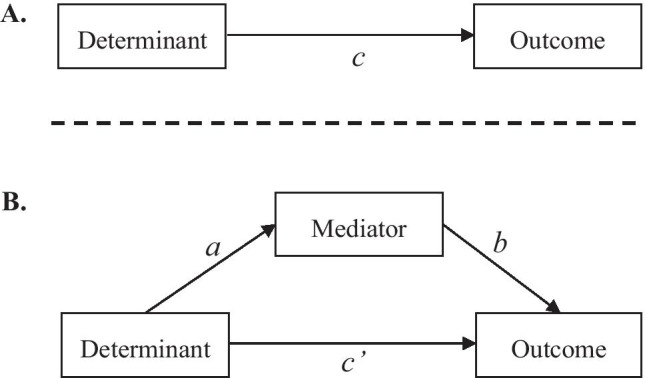
Path diagram of a single mediator model. **A** represents the total exposure-outcome effect (*c* path). **B** represents the indirect effect of the exposure on the outcome through the mediator (*a* and *b* paths) and the direct exposure-outcome effect (*c′* path)

Figure 1 represents a path diagram of a simple mediator model, in which the *c* path represents the total determinant-outcome effect, the *a* path represents the determinant-mediator effect, the *b* path represents the mediator-outcome effect, and the *c′* path represents the direct determinant-outcome effect (MacKinnon, 2008).

Traditionally, three linear regression equations are used to perform a mediation analysis (Baron & Kenny, 1986; Judd & Kenny, 1981):

1 $$Y={i}_{1}+cX+{\epsilon}_{1}$$

2 $$M={i}_{2}+aX+{\epsilon}_{2}$$

3 $$Y={i}_{3}+{c}^{\text{'}}X+bM+{\epsilon}_{3}$$

where in Eq. 1, the *c* coefficient is the total determinant-outcome effect. In Eq. 2, the *a* coefficient is the determinant-mediator effect. In Eq. 3, the *b* coefficient is the mediator-outcome effect adjusted for the determinant, and the *c′* coefficient is the direct determinant-outcome effect adjusted for the mediator. In all equations, *i*_*1*_, *i*_*2*_, and *i*_*3*_ are intercept terms, and *ε*_*1*_, *ε*_*2*_, and *ε*_*3*_ are residual terms.

The mediation analysis methodology underwent many advancements in recent years. In the 1980s, Judd and Kenny (1981) and Baron and Kenny (1986) described the causal steps method for mediation analysis, in which the presence of a mediated effect was determined based on the statistical significance of the coefficients estimated based on Eqs. 1–3. Later, the product-of-coefficients (*ab*) method and difference-in-coefficients (*c*–*c′*) method for estimating the indirect effect were described (MacKinnon & Dwyer, 1993). In this paper, we refer to these methods as “traditional mediation analysis.”

The most recent advancement in the mediation analysis methodology is the development of causal mediation analysis (Imai et al., 2010; Pearl, 2012; VanderWeele, 2015). This method stresses the importance of the no (unobserved) confounder assumptions and defines and estimates effects as the difference between two potential outcomes, providing controlled direct effect estimates and natural direct and indirect effect estimates that take into account determinant-mediator interaction. These causal estimators provide similar effect estimates as in traditional mediation analysis for mediation models with a continuous mediator and a continuous outcome, but not necessarily for other types of mediation models (MacKinnon et al., 2020; Pearl, 2012; Rijnhart et al., 2017, 2020; VanderWeele, 2015).

## Multiverse Analysis of a Mediation Analysis

The general goal of a multiverse analysis is to assess the robustness of the effect estimates against data analytical decisions (Simonsohn et al., 2020; Steegen et al., 2016). It helps to identify the most impactful decisions and thereby provides important information for the development of a more complete and precise research theory (Del Giudice & Gangestad, 2021; Steegen et al., 2016). Multiverse analyses may be applied in original studies or to assess the robustness of previously published results. A multiverse analysis generally consists of three steps (Simonsohn et al., 2020; Steegen et al., 2016). In the first step, the multiverse is determined by identifying all decision points and reasonable alternative decisions. In the second step, the data is analyzed across this multiverse. In the third step, the effect estimates are summarized and interpreted.

### Step 1: Identification of the Multiverse

In the first step, the decision points are identified, and all reasonable alternative decisions are determined before analyzing the data (Simonsohn et al., 2020). Decision points vary across studies and may be study-centric (e.g., exclusion criteria and missing data handling), variable-centric (e.g., variable transformations), or model-centric (e.g., the inclusion of interactions, random effects, and covariates in the statistical model) (Steegen et al., 2016). After the decision points are identified, a set of reasonable alternative decisions is determined for each decision point. These alternative decisions should be consistent with the underlying theoretical framework, statistically valid, and not redundant with other decisions in the multiverse (Simonsohn et al., 2020). In other words, the alternative decisions should reflect the arbitrary RDFs, but not include QRPs (Del Giudice & Gangestad, 2021; Wicherts et al., 2016). In contrast with conventional sensitivity analyses based on alternative decisions selected by the researcher, the goal of a multiverse analysis is to identify all decision points and *all* reasonable alternative decisions.

The decision points and alternative decisions may be summarized using a table (Steegen et al., 2016; Simonsohn et al., 2020) or an analytic decisions graph (Liu et al., 2020; Stern et al., 2019). It is important to provide rationales for the alternative decisions, as this increases transparency and helps readers understand why the alternative decisions reflect RDFs rather than QRPs (Simonsohn et al., 2020). Table 1 provides an overview of potential decision points and alternative considerations relevant for a mediation analysis. Some of the decision points in Table 1 are relevant for any type of analysis, while the *italicized* decision points and alternative considerations specifically apply to mediation analyses.

**Table 1:** Overview of potential decision points and alternative decisions for the multiverse analysis of a mediation analysis

Decision points	Alternative considerations
Determinant variable	Alternative operationalizations of the determinant, e.g., defining the variable differently based on the same measure or using an alternative measure of the same construct
Outcome variable	Alternative operationalizations of the outcome variable, e.g., defining the variable differently based on the same measure or using an alternative measure of the same construct
*Mediator variable*	*Alternative operationalizations of the mediator variable, e.g.,* *defining the variable differently based on the same measure or using an alternative measure of the same construct*
Confounder variables	Alternative operationalizations of the confounder variables, e.g., using different cut-off points for a binary or categorical confounder variable Varying sets of confounders of the determinant-outcome effect, *the determinant-mediator effect, and the mediator-outcome effect* Use of an alternative confounder adjustment method, e.g., inverse probability weighting
Moderator variables	Alternative or additional moderators of the determinant-outcome effect, *the determinant-mediator effect, and the mediator-outcome effect* *Assessment of determinant-mediator interaction*
Exclusion criteria	Varying sets of exclusion criteria, potentially varying from not excluding any participant to strict exclusion criteria
Missing data handling	Use of multiple imputation or full-information maximum likelihood
*Mediation analysis method*	*Use of causal mediation analysis if the original study used traditional analysis*
Type of regression models	Use of varying analysis techniques to estimate the outcome model and *mediator model,* e.g., log-linear regression instead of logistic regression
Functional form	Alternative functional form of the determinant-outcome effect, *the determinant-mediator effect, and the mediator-outcome effect*, e.g., using quadratic or cubic terms
*Determining the presence of a mediated effect*	*Based on the estimation of confidence intervals that take into account the skewed distribution of the indirect effect, e.g., distribution of the product, Monte Carlo, or bootstrap confidence intervals*
Unmeasured confounding	Assessment of the impact of various sets of unmeasured confounders of the determinant-outcome effect, *the determinant-mediator effect, and the mediator-outcome effect* using sensitivity analyses

Note: The decision points and alternative considerations specific to mediation analysis are *italicized*

The mediation-analysis–specific decisions include the operationalization of the mediator variable and the consideration of potential confounders and moderators of the determinant-mediator and mediator-outcome effects. The mediation analysis methodology underwent many advancements in recent years. Previously published mediation analyses might therefore be based on suboptimal methodology, which could be addressed in the multiverse analysis. For example, if the published study assessed the statistical significance of the indirect effect estimate using normal-theory–based confidence intervals, confidence intervals that take into account the skewed distribution of the indirect effect estimate (e.g., distribution of the product confidence intervals, Monte Carlo confidence intervals, and bootstrap confidence intervals) could be considered as an alternative, as these have higher power to detect a statistically significant indirect effect estimate (Mackinnon et al., 2004). If the published study applied traditional mediation analysis methods, causal mediation analysis may be used to inform alternative decisions. The causal effect estimation may differ from the traditional effect estimation for mediation models with non-continuous mediator variables and non-continuous outcome variables (Pearl, 2012; Rijnhart et al., 2020; VanderWeele, 2015). Furthermore, causal mediation analysis takes into account determinant-mediator interaction and provides sensitivity analyses for unmeasured confounders (Imai et al., 2010; MacKinnon et al., 2020; Pearl, 2012; VanderWeele, 2015). Detailed information on (the application of) causal mediation analysis can be found elsewhere (e.g., Imai et al. (2010), Pearl (2012), VanderWeele (2015), and Valente et al. (2020)).

### Step 2: Data Analysis

In the second step, the data is analyzed across the multiverse identified at the first step (Simonsohn et al., 2020; Steegen et al., 2016). This step also involves checking for redundancy among alternative decisions. Redundancy means that alternative decisions lead to the same data situation. For example, when multiple criteria for determining the confounder set lead to the same confounder set, then it will not be necessary to include all criteria.

Since a multiverse analysis involves multiple testing, type 1 error rates may be elevated. The significance level may be adjusted to account for this, but this may come at the cost of elevated type 2 error rates (Ranganathan et al., 2016). Instead, we advise to focus primarily on the patterns of the results and the absolute or relative effect sizes when interpreting the effect estimates.

### Step 3: Summarizing and Interpreting the Results

In the third step, the effect estimates yielded by the multiverse analysis are summarized and interpreted (Simonsohn et al., 2020; Steegen et al., 2016). Effect estimates and *p*-values can be summarized using kernel density plots, histograms, grids, or volcano plots (Patel et al., 2015; Steegen et al., 2016; Young & Holsteen, 2017). A downside of these reporting methods is that they do not provide insight into the impact of specific data analytical decisions on the magnitude and statistical significance of the effect estimates. Alternatively, Simonsohn et al. (2020) proposed the use of specification curves to plot the effect estimates against the data analytical decisions. A specification curve consists of two panels, the top panel displays the effect estimates, and the lower panel displays the combination of data analytical decisions that led to each effect estimate in the top panel. Based on mediation analysis, specification curves can be constructed for the direct, indirect, and total effect estimates. Finally, it is important to note that the interpretations of the effect estimates may differ across decisions (Del Giudice & Gangestad, 2021; Simonsohn et al., 2020). For example, when analyses are based on various scales for the determinant, mediator, or outcome variable or different sets of covariates.

## Data Example

We demonstrate the multiverse analysis of a mediation analysis using data from the Longitudinal Aging Study Amsterdam (LASA). This is a prospective cohort study aiming to assess the determinants, trajectories, and consequences of changes in physical, cognitive, emotional, and social functioning with aging. The cohort consists of a nationally representative sample of participants initially aged 55 to 84 years. The data collection has been ongoing since 1992/1993, with measurements every 3 years. Measurements consist of a main interview, a self-administered questionnaire, and a medical interview. Detailed information on the LASA study can be found in Hoogendijk et al. (2020).

We reanalyzed the mediation analyses originally published by Pluijm et al. (2001), who assessed to what extent the effects of age, change in body weight, lifestyle, chronic diseases, medication use, and hormonal indices on bone mineral density (BMD) are mediated by body composition, which was measured as fat mass and appendicular muscle mass. For this study, data were used from the 1995/1996 measurement wave (*n* = 2,545). Participants were excluded if no interview or dual-energy X-ray absorptiometry (DXA) data was available for the 1995/1996 measurement and if they had both hips replaced. Only people born in or before 1930 and living in Amsterdam and its vicinity were invited for a DXA scan. A total of 522 participants were eligible for the analyses. All analyses were carried out separately for females (*n* = 264) and males (*n* = 258).

The original study considered seventeen potential determinants of BMD, including age, change in body weight since age 25, lifestyle factors, chronic diseases, medication use, and hormonal indices. The original study results supported the hypothesis that fat mass is a mediator of the relation between weight change, walking activities, and sex hormone–binding globulin and BMD in women only. In our reanalysis, we assessed the robustness of the finding that fat mass mediates the relation between weight change and BMD in women against various data analytical decisions (a path diagram of the mediation model can be found in Supplementary Fig. S1). In the next section, we describe the decisions made in the original study and the alternative decisions included in the multiverse analysis.

### Decision Points and Alternative Decisions

 First, we identified the decision points based on the information in the original paper by Pluijm et al. (2001). Then, we determined all reasonable alternative decisions. The identified multiverse consisted of 108 direct and indirect effect estimates (i.e., 3 × 2 × 2 × 3 × 3 = 108), each for which we determined the presence of a mediated effect in two ways: based on the indirect effect with a confidence interval and based on the criteria in the original paper. Table 2 summarizes all data analytical decisions and alternative decisions for the data example. The decision points and alternative decisions are described in greater detail below.

**Table 2:** Overview of decision points and alternative decisions included in the multiverse analysis of the data example in which fat mass is investigated as a mediator of the relation between weight change and BMD

Decision points	Decisions included in the multiverse analysis
Determinant variable	1. Continuous (percentage change) 2. Categorical: increased weight versus stable weight 3. Categorical: decreased weight versus stable weight
Confounder variables	Set of confounders: 1. Height, age, smoking, alcohol use, and minutes of walking in past two weeks, sports in last two weeks, COPD, stroke, rheumatoid arthritis, and diabetes, corticosteroid use, estrogen use, SHBG, PTH, IGF-1, 25(OH)D, and Albumin 2. Height, age, smoking, alcohol use, and minutes of walking in past two weeks, sports in last two weeks, COPD, stroke, rheumatoid arthritis, and diabetes, corticosteroid use, estrogen use Consideration of confounders: 1. A priori adjustment based on theory 2. Based on ≥ 10% change in any effect estimate
Moderator variables	Moderation by age: 1. Based on all ages 2. Based on < 75 years of age 3. Based on ≥ 75 years of age
	Determinant-mediator interaction: 1. No assessment of determinant-mediator interaction 2. Estimation of pure natural direct effects and pure natural indirect effects 3. Estimation of total natural direct effects and total natural indirect effects
Determining the presence of a mediated effect	1. Based on causal steps and a proportion mediated of 20% or higher 2. Based on natural indirect effect estimates with 95% Monte Carlo confidence intervals

Note: Every first decision represents the decision made in the original study

#### Determinant

Change in body weight was computed as the percentage change in body weight between the self-reported lowest body weight since age 25 and the body weight measured in 1995/1996. Percentage change in body weight was treated as a continuous variable in the original study.

In the reanalysis of the data, we additionally treated change in body weight as a categorical variable, with the categories representing decreased weight (*n* = 22), stable weight (*n* = 43), and increased weight (*n* = 199). This categorical weight change variable was computed based on the Edwards-Nunnally index, which provides a categorization of change that is less sensitive to natural fluctuations and measurement error than the continuous percentage weight change variable, as it determines individual significant change based on the reliability (Cronbach’s alpha), mean, and standard error of the first weight measurement (Speer & Greenbaum, 1995). Informed by previous research (Stevens et al., 1990), we computed the Edwards-Nunally index based on a Cronbach’s alpha of 0.822. Participants for whom the Edwards-Nunally index did not indicate a significant increase or decrease in weight were classified as stable weight. We estimated two direct effects and two indirect effects based on the categorical determinant; one for increased weight versus stable weight and one for decreased weight versus stable weight.

#### Mediator and Outcome Variables

Fat mass (kg) was computed based on total body DXA measurements and was treated as a continuous variable in the original study. The DXA measurement of the hip was used to determine the BMD (mg/cm^2^) of the hip, which was also treated as a continuous variable in the original study. We did not make alternative decisions on the mediator and outcome variables for the reanalysis of the data.

#### Confounder Variables

In the original study, the associations between body weight, fat mass, and BMD were adjusted for height in centimeters, smoking status (never smoking, former smoker, and current smoker), average number of alcoholic consumptions per week, average number of minutes of walking outside the house per day, presence of chronic obstructive pulmonary diseases (COPD), presence of diabetes mellitus, history of stroke, presence of rheumatoid arthritis, use of corticosteroids (current and former versus never), use of estrogens (among women only; current and former versus never), log-transformed SHBG, log-transformed parathyroid hormone (PTH), serum 25-hydroxyvitamin D (25(OH)D), insulin-like growth-factor 1 (IGF-1), and albumin. However, the hormonal factors, SHBG, PTH, 25(OH)D, IGF-1, and albumin, might be influenced by fat mass rather than vice versa (Pluijm et al., 2001). Due to the cross-sectional nature of the data, the causal order of fat mass and the hormonal factors remained unclear. Therefore, we alternatively adjusted the analyses for the aforementioned set of confounders excluding these hormonal factors. In the original study, all analyses were adjusted for the a priori specified set of confounder variables. To preserve power, we alternatively adjusted the analyses for variables that caused a minimum of 10% change in any of the *a*, *b*, and *c′* path estimates (i.e., (adjusted beta – unadjusted beta)/unadjusted beta × 100).

#### Moderator Variables

In the reanalysis of the data, we considered age as a potential moderator of the paths in the mediation model. Age was treated as a binary variable to estimate the effects for the young-old (i.e., < 75 years) and the old-old (i.e., ≥ 75 years) separately (Orimo et al., 2006). Determinant-by-age and mediator-by-age interaction terms were added to the estimated regression models based on Eqs. 2 and 3. Subsequently, the effects for the young-old and old-old were estimated based on the simple slopes from these equations (Aiken & West, 1991).

#### Statistical Analyses

In the original study, multiple linear regression analysis was used to perform the mediation analysis, and the presence of a mediated effect was determined based on the causal steps criteria and a proportion mediated of 20% or larger. No indirect effect estimates were reported in the original study, and the statistical significance of the mediated effect was also not assessed. In our reanalysis, we quantified the mediated effect by estimating natural indirect effects based on causal mediation analysis with corresponding 95% Monte Carlo confidence intervals. We accounted for potential determinant-by-mediator interaction by adding determinant-by-mediator interaction terms to Eq. (3). Subsequently, we estimated pure and total natural direct and indirect effects (MacKinnon et al., 2020; VanderWeele, 2015). The pure natural direct effect was estimated as the direct effect of weight change on BMD when holding each woman’s fat mass constant at the value that would have been observed if that woman did not change in weight. The total natural direct effect was estimated as the direct effect of weight change on BMD when holding each woman’s fat mass constant at the value that would have been observed if that woman did change in weight. The pure natural indirect effect was estimated as the indirect effect of weight change on BMD through fat mass when the determinant was held constant at the no weight change value. The total natural indirect effect was estimated as the indirect effect of weight change on BMD through fat mass when the determinant was held constant at the weight change value. We did not apply alternative missing data handling strategies, as the percentage of missing values was small (i.e., it ranged between 0% and 6.8%) (Bennett, 2001).

### Multiverse Analysis

We first assessed whether any of the identified 108 conditions were redundant. Specifically, we assessed whether any of the confounder sets based on ≥ 10% change in any of the effect estimates were redundant with the a priori specified confounder sets. For all non-redundant conditions, we then estimated the direct, indirect, and total effects. Effect estimates were considered statistically significant when p < 0.05. All analyses were performed using Stata statistical software release 14.1 (StataCorp, 2016). The specification curves were plotted using Stata code provided by Simonsohn et al. (2020). The dataset with the effect estimates and Stata code for the specification curves are provided in the supplementary materials.

## Results

First, to check for redundancy among the confounder sets, we determined the confounder sets based on ≥ 10% change in any of the effect estimates based on the continuous and categorical weight change variables. For the mediator models based on the continuous weight change variable, we identified smoking, estrogen use, IGF-1, 25(OH)D, albumin, height, ln-SHBG, and ln-PTH as confounders. For the mediator models based on the categorical weight change variable, we identified age, smoking, alcohol use, walking, COPD, stroke, estrogen use, IGF-1, 25(OH)D, albumin, height, ln-SHBG, and ln-PTH as confounders. The confounder sets determined based on ≥ 10% change in the effect estimates differed from the a priori determined confounder sets and were therefore not redundant.

The multiverse analysis resulted in 108 indirect and direct effect estimates and 36 total effect estimates. Based on the criteria from the original paper, i.e., the causal steps criteria and a proportion mediated of 20% or larger; fat mass mediated the relation between weight change and BMD in 55.6% of the conditions. Based on the statistical significance of the indirect effect estimates, fat mass mediated the relation between weight change and BMD in 64.8% of the conditions. This percentage is higher than the percentage based on the criteria from the original paper for two reasons. First, the statistical significance of the indirect effect estimates is not affected by the non-significance of the total effect estimates in inconsistent mediation models (i.e., when the indirect effect estimates are positive and the direct effect estimates are negative) (MacKinnon, 2008). Second, some indirect effect estimates were statistically significant while the corresponding proportion mediated did not exceed 20%.

**Fig. 2:**
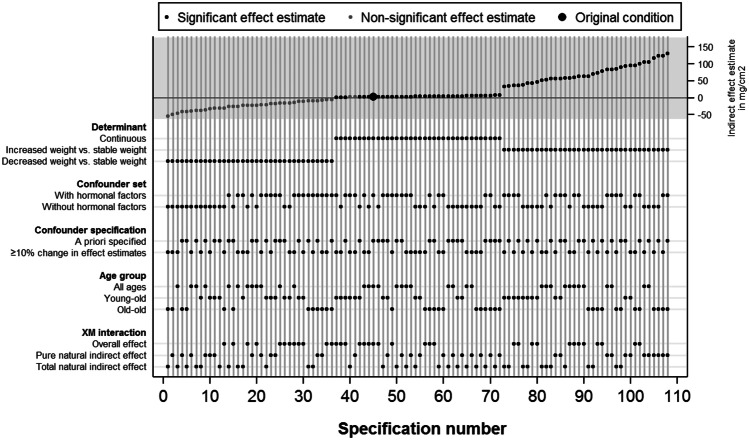
Specification curve of the indirect effect estimates of weight change on bone mineral density (mg/cm^2^) through fat mass (kg)

Figure 2 summarizes the indirect effect estimates in a specification curve, with the upper panel displaying the indirect effect estimates in ascending order, and the dots in the lower panel indicating the data analytical decisions corresponding to each indirect effect estimate in the upper panel. For example, the lowest indirect effect estimate corresponded to the condition in which the total natural indirect effect was estimated for women in the old-old group with a decreased weight versus women in the old-old group with a stable weight, adjusted for the confounder set based on ≥ 10% change without hormonal factors.

The indirect effect estimates ranged between − 54.1 mg/cm^2^ and 137.0/cm^2^. The indirect effect estimate based on the condition from the original paper, denoted by the large dot in Fig. 2, equaled 2.92 mg/cm^2^, indicating that for a one percentage increase in weight, women on average had a 2.92 mg/cm^2^ higher BMD through an increase in fat mass. The corresponding 95% Monte Carlo confidence interval indicated that this effect estimate was statistically significant. Negative indirect effect estimates were only observed for conditions in which women with a decreased weight were compared to women with a stable weight, indicating that women with a decreased weight on average have a lower BMD than women with a stable weight through a decrease in fat mass. The positive indirect effect estimates based on the continuous determinant and the categorical determinant comparing women with increased weight to women with a stable weight indicate that women with an increased weight on average had a higher BMD than people with a stable weight through an increase in fat mass.

Like in the original paper, the direct effect estimates in most conditions were negative (86.1%), and most were not statistically significant (91.7%). In contrast, the total effect estimates in most conditions were positive (66.7%) and most were statistically significant (55.6%). Specification curves for the direct and total effect estimates can be found in Supplemental Figs. S2 and S3, respectively).

To summarize, the multiverse analysis showed that the direct, indirect, and total effect estimates were generally in line with the research hypotheses and therefore robust against alternative data analytical choices concerning the determinant, confounder set, confounder specification, age interactions, and determinant-mediator interaction. Therefore, the multiverse analysis results support the hypothesis that fat mass is a mediator of the relation between weight change and BMD.

## Discussion

The aim of this paper was to provide an overview and worked example of the use of multiverse analysis to assess the robustness of the effect estimates from a mediation analysis. To our knowledge, this is the first description of multiverse analysis as a method to assess the robustness of mediation analysis results. In Table 1, we demonstrated that the multiverse of a mediation analysis consists of more decision points than the multiverse of a bivariate analysis. In our data example, we demonstrated the use of specification curves to visualize the impact of the original and alternative decisions from the multiverse on the magnitude and statistical significance of the direct, indirect, and total effect estimates. This information subsequently can be used to refine the underlying research theory and inform replication studies.

We demonstrated the application of multiverse analysis using a data example from the LASA study. We assessed the robustness of the effect estimates from a previously published mediation analysis in which fat mass was investigated as a mediator of the relation between weight change and BMD. The effect estimates in our data example were generally robust against alternative data analytical decisions and in line with the underlying research theory. In practice, a multiverse analysis might alternatively reveal that effect estimates are not robust against alternative data analytical decisions. For example, suppose that a mediation analysis is performed to assess the effectiveness of an intervention aimed at preventing overweight through stimulating physical activity. Suppose that the multiverse analysis indicates that the magnitude or statistical significance of the indirect effect estimate is sensitive to the definition of physical activity (e.g., including or excluding low-intensity activities). In such a situation, the information from the multiverse analysis can be used to refine the underlying research theory (Del Giudice & Gangestad, 2021; Steegen et al., 2016).

In this paper, we provided an overview of decision points relevant for mediation analyses. However, the decision points and alternative considerations described in this paper are by no means exhaustive. Additional decision points might be relevant for more complex mediation models, such as longitudinal mediation models (MacKinnon, 2008; Maxwell & Cole, 2007). Furthermore, although multiverse analysis increases the transparency of the research process, the identification of the decision points and alternative decisions remains a subjective process (Simonsohn et al., 2020; Steegen et al., 2016). This subjectivity was illustrated in the studies by Gangestad et al. (2019) and Stern et al. (2019), who identified different multiverses while addressing the same research question using the same data. Despite this subjectivity, multiverse analyses do offer more transparency than when only one model is reported, and with the accumulation of knowledge and methodological developments over time, new decisions can always be added to the multiverse (Simonsohn et al., 2020; Steegen et al., 2016; Young & Holsteen, 2017).

Multiverse analysis has some important strengths. First, multiverse analysis is a relatively cost-effective method to assess the robustness of published study results against arbitrary RDFs, as it does not require the collection of new data (Nuijten et al., 2018). Second, by performing analyses across various combinations of data analytical decisions, a multiverse analysis takes full advantage of the original data. Furthermore, the data sharing initiatives supported by an increasing number of journals enable researchers to perform multiverse analyses of published research results before trying to replicate results (Gewin, 2016). In addition to first reproducing the effect estimates in the original study, multiverse analysis has the potential to become an important step before performing a replication study.

Despite the advantages of multiverse analysis, its uptake in empirical studies remains low. A first potential reason for this low uptake is that there is only little guidance available on how to perform and report multiverse analyses (Dragicevic et al., 2019; Liu et al., 2020). By describing and demonstrating the steps involved in a multiverse analysis of a mediation analysis, this study aimed to stimulate the uptake of multiverse analysis as a method to assess the robustness of mediation analysis results. A second potential reason for the low uptake is that a multiverse analysis is more time-consuming than a single analysis (Liu et al., 2020). The time investment could be reduced if parts of the analyses could be automated. The MROBUST module in Stata is an example of a module that automates multiverse analysis, as it allows the users to estimate models across various combinations of data analytical decisions based on only one line of code (Young & Holsteen, 2016). However, this package is limited to bivariate analyses and therefore cannot be used for mediation analyses. Future studies could focus on the development of software for the automation of multiverse analyses of more complex analyses. Another strategy that has been proposed to reduce the time investment is the analysis of a random subsample of the identified multiverse conditions (Simonsohn et al., 2020). However, methodological studies still need to be undertaken to investigate what percentage of the identified multiverse conditions should be included in such a random sample and what random sampling technique should be applied to ensure valid results.

Another important topic for future research is the accuracy of summary measures computed based on the distribution of effect estimates yielded by a multiverse analysis. Examples of such summary measures are the mean effect estimate with a corresponding significance test based on a standard error for this mean effect estimate (Young & Holsteen, 2017), and the median effect estimate with a corresponding bootstrap confidence interval (Simonsohn et al., 2020). These two methods assume that the distribution of effect estimates can be summarized using either the mean or median effect estimate, respectively. However, various distributions of the effect estimates were observed in previous multiverse analyses, including multimodal distributions (see e.g., Young & Holsteen, 2017), indicating that the mean and median may not always be accurate summary statistics. Therefore, the development of accurate summary statistics is an important avenue for future research.

## Conclusion

Multiverse analysis is a useful method to assess the robustness of the direct, indirect, and total effect estimates from a mediation analysis against arbitrary RDFs. Specification curves can be used to visualize the impact of various combinations of data analytical decisions on the magnitude and statistical significance of the direct, indirect, and total effect estimates. The results from a multiverse analysis can inform future replication studies and help refine the underlying research theory.

## Supplementary Information

Below is the link to the electronic supplementary material.

Supplementary file1 (DOCX 579 KB)

Supplementary file2 (DO 12 KB)

Supplementary file3 (DTA 23 KB)

## Data Availability

The raw data used in this publication are freely available for replication purposes and can be obtained by submitting a research proposal to the LASA Steering Group, using a standard analysis proposal form that can be obtained from the LASA website: www.lasa-vu.nl. The LASA Steering Group will review all data requests to ensure that proposals for the use of LASA data do not violate privacy regulations and are in keeping with the informed consent that is provided by all LASA participants.
