# Supplementary material for: Assessing the Robustness of Mediation Analysis Results Using Multiverse Analysis
Source: Prev Sci. 2021 Jul 16;23(5):821–31. doi: 10.1007/s11121-021-01280-1 (PMC9283158; doi:10.1007/s11121-021-01280-1)
Supplement: Supplementary file 1 — Supplementary file1 (DOCX 579 KB) [file 11121_2021_1280_MOESM1_ESM.docx]

**Supplementary figures for “Assessing the robustness of mediation analysis results using multiverse analysis” by Rijnhart et al.**

| Bone mineral density  Fat mass  Weight change |
| --- |
| **Figure 1**  *Path diagram of the data example in which fat mass is hypothesized as a mediator of the relation between weight change and bone mineral density.* |

| 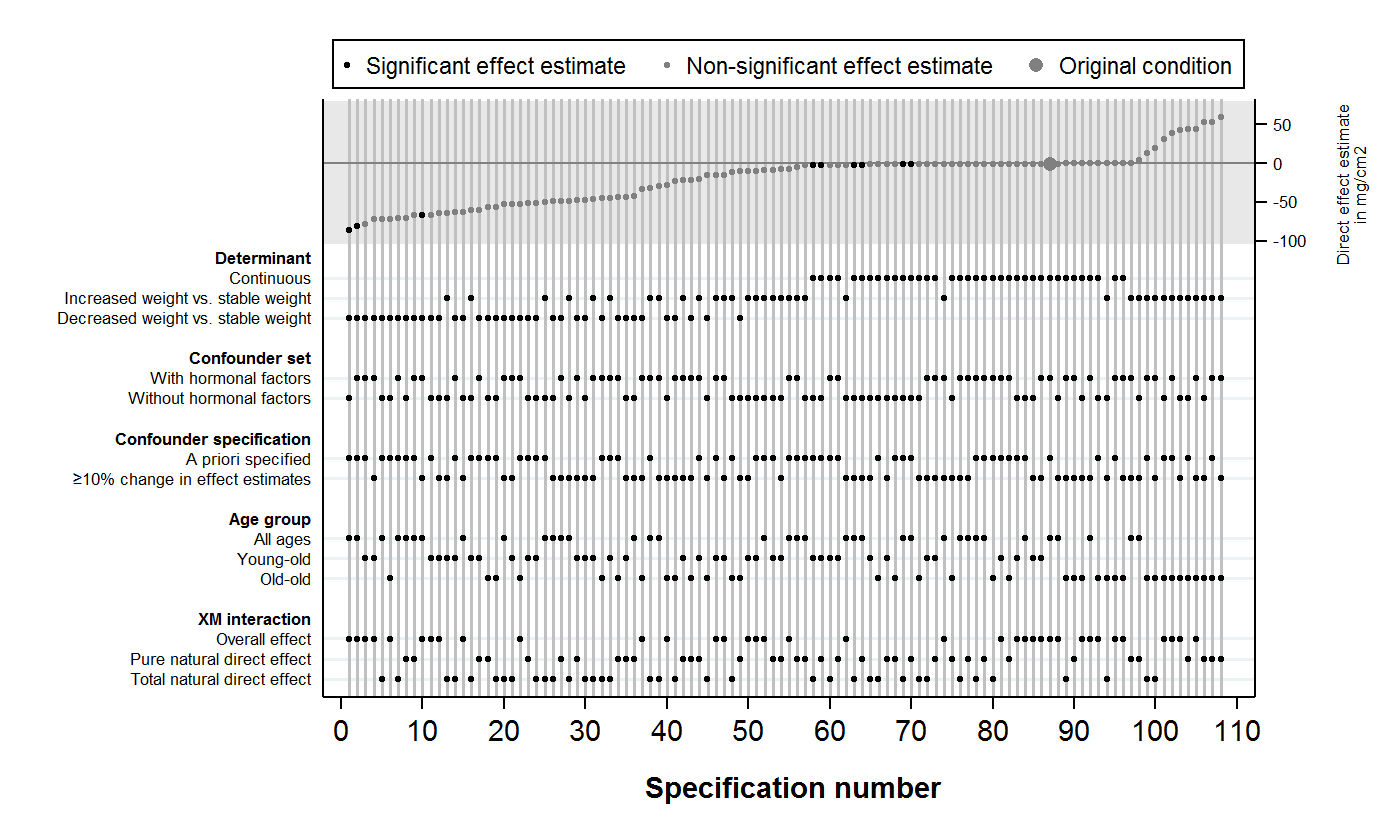 |
| --- |
| **Figure S2**  *Specification curve of the direct effect estimates of weight change on bone mineral density (mg/cm^2^)* |

| 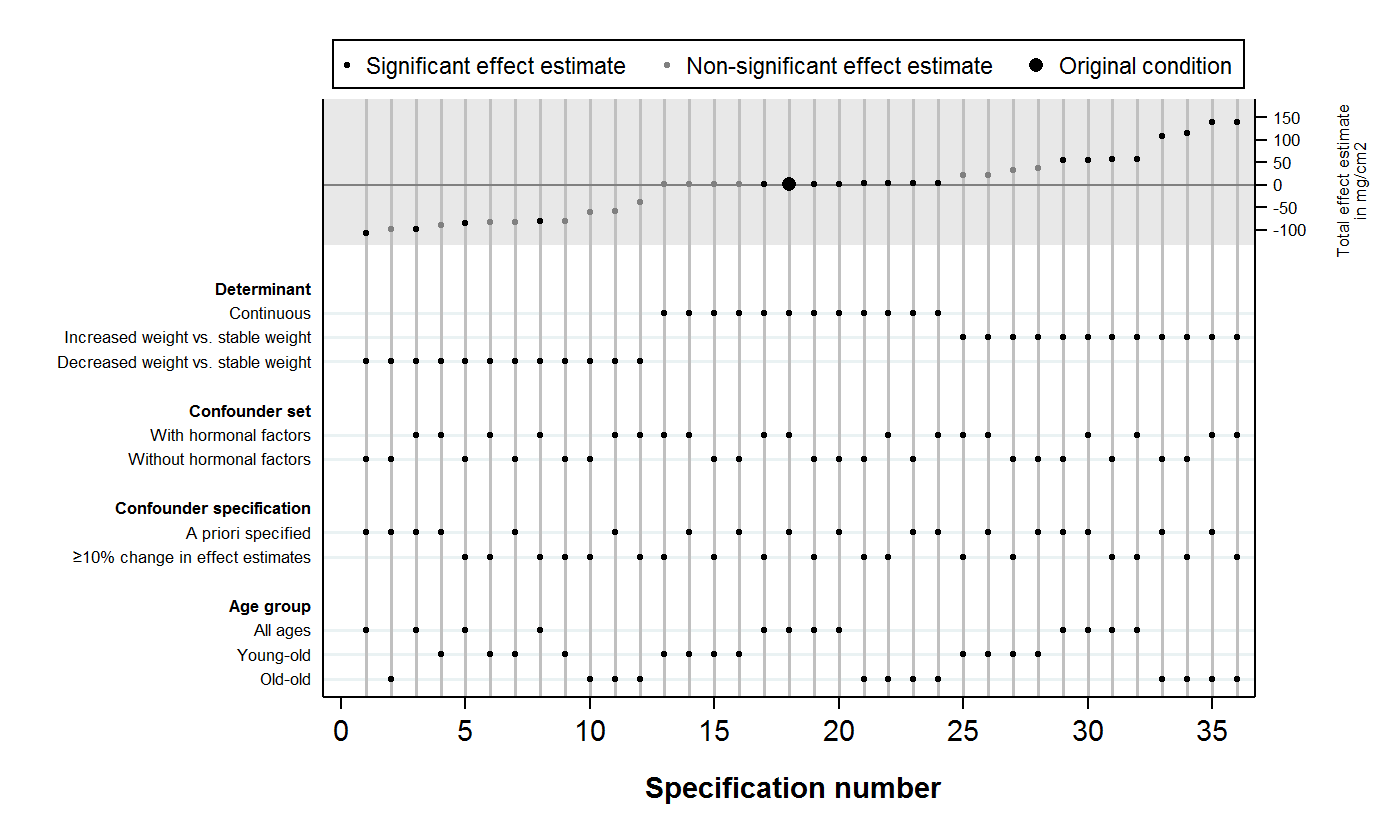 |
| --- |
| **Figure S3**  *Specification curve of the total effect estimates of weight change on bone mineral density (mg/cm^2^)* |
